# SF3B1 modulators affect key genes in metastasis and drug influx: a new approach to fight pancreatic cancer chemoresistance

**DOI:** 10.20517/cdr.2021.61

**Published:** 2021-10-08

**Authors:** Ornella Randazzo, Stella M. Cascioferro, Camilla Pecoraro, Widad Ait Iddouch, Amir Avan, Barbara Parrino, Daniela Carbone, Ugo Perricone, Godefridus J. Peters, Patrizia Diana, Elisa Giovannetti

**Affiliations:** ^1^Department of Medical Oncology, Cancer Center Amsterdam, Amsterdam UMC, VU University Medical Center, Amsterdam 1081 HV, The Netherlands.; ^2^Dipartimento di Scienze e Tecnologie Biologiche Chimiche e Farmaceutiche (STEBICEF), Università degli Studi di Palermo, Palermo 90133, Italy.; ^3^Metabolic Syndrome Research Center, Mashhad University of Medical Sciences, Mashhad 91886-17871, Iran.; ^4^Cancer Research Center, Mashhad University of Medical Sciences, Mashhad 91886-17871, Iran.; ^5^Student Research Committee, School of Medicine, Mashhad University of Medical Sciences, Mashhad 91886-17871, Iran.; ^6^Drug Discovery Unit, Fondazione Ri.MED, Palermo 90128, Italy.; ^7^Department of Biochemistry, Medical University of Gdansk, Gdansk 80-210, Poland.; ^8^Cancer Pharmacology Lab, AIRC Start Up Unit, Fondazione Pisana per la Scienza, Pisa 56124, Italy.; ^#^Authors contributed equally.

**Keywords:** Pancreatic ductal adenocarcinoma, gemcitabine, indole derivatives, anti-proliferative activity, anti-migratory activity, SF3B1, RON, hENT1

## Abstract

**Aim:** Because mutations of splicing factor 3B subunit-1 (SF3B1) have been identified in 4% of pancreatic ductal adenocarcinoma (PDAC) patients, we investigated the activity of new potential inhibitors of SF3B1 in combination with gemcitabine, one of the standard drugs, in PDAC cell lines.

**Methods:** One imidazo[2,1-*b*][1,3,4]thiadiazole derivative (IS1) and three indole derivatives (IS2, IS3 and IS4), selected by virtual screening from an in-house library, were evaluated by the sulforhodamine-B and wound healing assay for their cytotoxic and antimigratory activity in the PDAC cells SUIT-2, Hs766t and Panc05.04, the latter harbouring the SF3B1 mutations. The effects on the splicing pattern of proto-oncogene recepteur d’origine nantais (RON) and the gemcitabine transporter human equilibrative nucleoside transporter-1 (hENT1) were assessed by PCR, while the ability to reduce tumour volume was tested in spheroids of primary PDAC cells.

**Results:** The potential SF3B1 modulators inhibited PDAC cell proliferation and prompted induction of cell death. All compounds showed an interesting anti-migratory ability, associated with splicing RON/ΔRON shift in SUIT-2 cells after 24 h exposure. Moreover, IS1 and IS4 potentiated the sensitivity to gemcitabine in both conventional 2D monolayer and 3D spheroid cultures, and these results might be explained by the statistically significant increase in hENT1 expression (*P *< 0.05 *vs*. untreated control cells), potentially reversing PDAC chemoresistance.

**Conclusion:** These results support further studies on new SF3B1 inhibitors and the role of RON/hENT1 modulation to develop effective drug combinations against PDAC.

## INTRODUCTION

Pancreatic ductal adenocarcinoma (PDAC) is one of the most lethal cancers in the world. The survival rate has increased in recent years, and double-digit survival rates are increasingly seen, but epidemiological studies also report a rising incidence^[1,2]^. Thus, PDAC is projected to become the second leading cause of cancer-related death by 2030^[3]^. This grim future has multifactorial causes. There are no tools for prevention, and early diagnosis of PDAC is complicated. Most patients are diagnosed when the tumour has already spread throughout the body due to the lack of early symptoms and specific biomarkers^[4,5]^. The treatment options for PDAC are also relatively limited. The only current curative treatment at the moment is surgical resection, which is possible in only 20% of patients. Moreover, this treatment has a high complication rate and recurrence is often seen^[6]^. The standard of care treatment is chemotherapy, using polychemotherapy regimens or gemcitabine monotherapy^[7]^. Gemcitabine, approved by the Food and Drug Administration in 1996, was the standard of care in the treatment of locally advanced and metastatic PDAC for over two decades. A better efficacy was found for various chemotherapy combinations such as FOLFIRINOX [5-fluorouracil, folinic acid (leucovorin), irinotecan, oxaliplatin] and gemcitabine plus nab-paclitaxel (GEM-NAB, Abraxane®)^[8]^.

Most PDAC cases are characterised by inherent or acquired chemoresistance. This resistant behaviour is determined by multiple cellular-autonomous factors, such as reduced expression of key drug transporters, and/or by different components of the tumour microenvironment (TME)^[9,10]^.

Recent studies suggest that alternative splicing (AS) deregulation plays a pivotal role in tumorigenesis and cancer drug resistance^[11,12]^. Aberrant splicing has been shown to occur in genes involved in drug metabolism, including transporters responsible for drug uptake. In this regard, a well-known example of aberrant splicing is the exon 13 skipping in the *SLC29A1* gene (solute carrier family 29 member 1) which encodes the human equilibrative nucleoside transporter-1 (hENT1)^[13]^. This splicing alteration is due to an intronic mutation which leads to a reduction in the expression and uptake of another cytidine analogue, cytarabine^[11,14]^.

Drug resistance is also associated with alterations in genes that regulate apoptosis, often generating proteins with antagonistic functions (e.g., BCL-X and MCL-1) or migration (e.g., RON), favouring invasion and metastasis. Noteworthy, the pre-mRNA splicing process is also involved in the regulation of the DNA damage repair, influencing with high probability the resistance to therapy^[10,11,14,15]^.

Additionally, aberrations directly affect splicing regulation, and it has recently been demonstrated that somatic mutations of splicing factor genes are common in not only hematopoietic neoplasms but also solid tumours including PDAC^[16]^. The splicing factor 3B subunit-1 (SF3B1), which is involved in the branch site recognition during the pre-mRNA splicing process, is the most frequently mutated RNA splicing factor gene in cancer, and mutations in the HEAT domain of the *SF3B1* gene have been detected in 4% of PDAC patients^[12]^.

Against this background, and given the central role of AS in cancer, targeting this process is considered a potential therapeutic approach. Pre-clinical studies have shown potential in the modulation of splicing in cancer cells via small molecules targeting SF3B1^[11]^, namely pladienolide B (PB), spliceostatin A and herboxidiene, which interfere with the splicing modulation^[17]^. Two synthetic analogues of PB, E7107 and H3B-8800 (orally available small molecule), are the only SF3B1 modulators in clinical trials^[18]^. Of note, one patient with acinar pancreatic carcinoma and hepatic metastases had a confirmed partial response lasting eight months during the phase I trial testing E7107, but severe ophthalmologic disturbances halted further clinical development of this drug^[19]^.

As mentioned above, splicing modulation represents an innovative and interesting therapeutic strategy in the fight against cancer.

Preclinical studies revealed that low-dose splicing modulators are synergistic in combination with conventional anticancer agents^[20,21]^. We previously demonstrated that modulation of splicing in cancer cells was an effective therapy in an *in vivo* model, both as a monotherapy with direct inhibitors of SF3B1 and in combination with other anticancer agents, with acceptable toxicity^[11]^. This combination could expand the therapeutic window of the splicing modulators.

Therefore, investigations on new molecules that could target aberrant splicing in PDAC are warranted. In the present study, we performed structural computational studies and virtual screening of compounds available in an in-house molecular library, and we selected some indole derivatives to evaluate their antitumour activity in appropriate preclinical models of PDAC and their potential to fight molecular mechanisms underlying PDAC chemoresistance. In particular, we used the epithelial and mesenchymal cells SUIT-2 and Hs766t, as well as Panc05.04 cells, carrying the *SF3B1* mutations p.Q699H and p.K700E.

Remarkably, heterocyclic compounds play a pivotal role in the field of drug design because the insertion of a heterocyclic moiety into a molecule can modulate drug properties such as potency and selectivity through bioisosterism, lipophilicity, polarity and aqueous solubility^[22]^. Among heterocyclic compounds, indoles have been investigated extensively due to their interesting versatility^[23]^. Many natural and synthetic derivatives of indoles have indeed shown a wide spectrum of pharmacological properties including antibacterial^[24-27]^, antifungal^[28,29]^, anti-inflammatory^[30]^, antihistamine^[31]^, anticholinesterase, antioxidant^[32]^, anti-diabetic^[33]^, antiviral^[34]^ and anticancer activities^[35,36]^.

The promising results obtained in our previous studies concerning the anticancer properties of imidazo[2,1-*b*][1,3,4]thiadiazole and indole scaffolds^[25,37-40]^ prompted us to explore the biological activity of the selected compounds alone and in combination with gemcitabine. Gemcitabine still represents the cornerstone of PDAC treatment and in preclinical models of peritoneal mesothelioma we observed that our imidazo[2,1-*b*][1,3,4]thiadiazole derivatives potentiated its antiproliferative effects^[40]^. Since these results were associated with increased expression of hENT1, which plays a key role in the uptake and cytotoxicity of gemcitabine^[41]^, in the present study, we also focused on the effect of AS on hENT1 expression in order to bypass one of the most important mechanism involved in the resistance to gemcitabine.

## METHODS

### Ligand preparation and protein preparation

Both ligands to be screened and co-crystallised ligands within the Protein Data Bank (PDB) structures were optimised using EpiK tool to energetically minimise their structure and generate protomers and tautomers at pH 7.4 ± 0.5^[42,43]^.

The 3D structures of the SF3B complex were downloaded from the PDB^[44]^ and imported into the Schrödinger suite to optimise the structure by using the “Protein preparation” tool^[42]^. The bond orders for untemplated residues were assigned and hydrogens were added to the structure. Water molecules beyond 5.0 Å from any of the HET groups, including ions, were deleted. Finally, PROPKA^[45]^ was run under pH 7.0 to optimise side chain states.

### Pharmacophore creation and screening

LigandScout^[46,47]^ software was employed to create the pharmacophore model and find the common feature between the two PDB structures by using the common pharmacophore map for virtual screening. The pharmacophore model was created, using the PDB coordinate of the ligand-protein complex (PDB IDs: 5ZYA and 6EN4). Starting from the two pharmacophore maps, only the common features were retained to be used for further studies. In the screening module, the “pharmacophore fit-score” was used as scoring function and “match all query features” was chosen as screening mode. The selected retrieval mode was “get best matching conformation”.

### Docking

The docking grid was generated using Glide software^[48]^. The scaling factor was set at 1.0 Å with a partial charge cut-off of 0.25, and the co-crystalised ligand was chosen as grid centroid. Molecular docking was carried out using Glide software^[48]^ by Schrödinger (release 2018-4). The van der Waals radii scaling factor for ligands to be screened was set as 0.8, with a partial charge cut off by 0.15. The ligands were considered as flexible, and Epik state penalties were considered as docking score. The in-house compounds library was screened in standard precision mode. Molecules were then ranked based on the docking score.

### Drugs and chemicals

The imidazo[2,1-*b*][1,3,4]thiadiazole derivative IS1 and the indole derivatives IS2, IS3 and IS4 were synthesised at the Department of Pharmacy, University of Palermo, Italy, following the synthetic procedures previously described^[39,49]^. The compounds were dissolved in dimethyl sulfoxide (DMSO), as reported previously^[41]^. Gemcitabine was kindly provided by Eli Lilly Corporation (Indianapolis, IN, USA) and dissolved in sterile water. Cell medium and newborn calf serum (NBCS) were from Gibco (Gaithersburg, MD, USA), while penicillin (50 IU mL^-1^) and streptomycin (50 µg mL^-1^) were from Lonza (Switzerland). Insulin-transferrin-selenium 100× was from Gibco (Grand Island, NY, USA) and PB was purchased from Cayman Chemical (Ann Arbor, MI, USA). All other chemicals were purchased from Sigma-Aldrich (Zwijndrecht, The Netherlands).

### Cell lines and culture conditions

The PDAC cell lines SUIT-2 and Hs766t were purchased from the American Type Culture Collection (ATCC, Manassas, VA), while the Panc05.04 cell line was a generous gift from Dr Eric Eldering (Department of Experimental Immunology, AMC, The Netherlands). SUIT-2 is a mesenchymal tumour cell line derived from a metastatic liver tumour of human pancreatic carcinoma. It produces and releases two tumour markers, carcinoembryonic antigen and carbohydrate antigen 19-9 (CA19-9), in culture *in vitro* and in nude mice *in vivo*^[50]^. Hs766t is an epithelial cell line isolated by R. Owens *et al*.^[51]^ in 1973 from a pancreatic carcinoma metastatic to a lymph node (ATCC® catalogue number HTB-134™). Panc05.04 is a pancreatic adenocarcinoma epithelial cell line derived, in 1995, from a primary tumour resected from the head of the pancreas of a woman with PDAC (ATCC® catalogue number CRL-2557™). The PDAC-3 primary culture cells were obtained from patients undergoing pancreatoduodenectomy, as described previously^[52]^.

PDAC-3, SUIT-2 and Hs766t cells were cultured in RPMI-1640 (Roswell Park Memorial Institute 1640) and DMEM (Dulbecco’s Modified Eagle’s Medium) medium, respectively, supplemented with 10% NBCS and 1% penicillin/streptomycin. Panc05.04 cells were cultured in RPMI-1640 supplemented with 20% NBCS, 1% penicillin/streptomycin and insulin-transferrin-selenium 100×. The cells were kept in a humidified atmosphere of 5% CO_2 _and 95% air at 37 ℃ and harvested with trypsin-EDTA (Ethylenediaminetetraacetic acid) in their exponential phase cultures. The cells were tested for mycoplasma monthly using the MycoAlert Mycoplasma Detection Kit (Westburg, Leusden, The Netherlands).

### Evaluation of cell growth inhibition by the sulforhodamine-B assay

*In vitro* chemosensitivity was assessed with the sulforhodamine-B (SRB) assay, as reported previously^[53,54]^. The SUIT-2 and Hs766t cells were seeded in triplicate in 96-well flat bottom plates at their optimal seeding concentration of 3-5 × 10^3^ cells in 100 µL/well for both cell lines. They were incubated overnight at 37 ℃ with 5% CO_2_ to ensure cells adhesion creating a confluent monolayer. Cells were treated in triplicate with 100 µL of drugs dissolved in DMSO at different concentrations in the nano- and micro-molar range and incubated at 37 ℃ with 5% CO_2_ for 72 h. Thereafter, the cells were fixed with 25 µL of cold 50% trichloroacetic acid for at least 60 min at 4 ℃. Then, the medium was removed, and the plates were gently washed five times with tap water, dried at room temperature overnight and stained with 50 µL of 0.4% (w/v) SRB solution in 1% acetic acid for 15 min at room temperature (RT). The plates were gently washed four times with 1% acetic acid and dried at RT for a minimum of 6 h. After adding 150 µL of tris(hydroxymethyl)aminomethane solution, the plates were gently mixed for 2-3 min at 350-400 rpm on a plate shaker. The optical density (OD) was spectrophotometrically read at wavelengths of 490 and 540 nm on a plate reader (BioTek Instruments Inc., Winooski, VT). Cell growth inhibition was calculated as the percentage of drug treated cells *vs*. vehicle-treated cells (“untreated cells or control”) OD (corrected for OD before drug addiction, “Day 0”). The 50% inhibitory concentration of cell growth (IC_50_) was calculated by non-linear least squares curve fitting (GraphPad PRISM, Intuitive Software for Science, San Diego, CA).

Since gemcitabine is commonly used (in monotherapy or within polychemotherapy regimens) for the treatment of PDAC patients and our previous studies in preclinical models of mesothelioma showed that thiadiazole derivatives potentiated gemcitabine effects^[40]^, we evaluated the cytotoxic activity of the most promising compounds (IS1 and IS4) in combination with gemcitabine. For these studies, we used the above-described SRB assay exposing cells to IC_50_ values of the experimental compounds, added to IC_25_ values of gemcitabine, for 72 h, as described previously^[40]^.

### Evaluation of cell death by trypan blue assay

The *in vitro* sensitivity to the most promising compounds (IS1 and IS4) was also assessed for the PDAC cell line Panc05.04 carrying two endogenous SF3B1 mutations: p.Q699H and p.K700E. Of note, these cells have a duplication time above 36 h and are therefore less suitable for the assessment of cytotoxic activity in 96-well plates with the SRB assay. Therefore, we used a trypan blue assay, as described below. The Panc05.04 cells were seeded in a 6-well flat bottom plate in a volume of 1 mL at the density of 2 × 10^4^ cells/well. They were incubated overnight at 37 ℃ with 5% CO_2_ to create a confluent monolayer and treated with 1 mL of drug dissolved in DMSO at concentrations ranging from 0.1 to 10 µM. After 96 h of treatment, the old medium was removed and the cells were washed twice with phosphate-buffered saline (PBS). Cells were harvested with trypsin-EDTA and incubated for 15 min at 37 ℃ with 5% CO_2_. After the addition of the new medium, the cells were resuspended and 10 µL of the cell suspension was harvested into a sterile Eppendorf. Noteworthy, only dead cells are coloured, since healthy living cells exclude trypan blue and are not coloured in this assay. Specifically, trypan blue is unable to penetrate the intact cell membrane of living cells. On the contrary, dead cells have a peculiar blue colour due to the absorption of the dye that crosses the compromised cell membrane. Trypan blue (10 µL) and 10 µL of the mixture for each Eppendorf were transferred to a cell counting slide. The percentage of viable cells *vs*. non-viable cells was determined using the LUNA II™ Automated Cell Counter according to the manufacturer’s protocol (Westburg, Leusden, The Netherlands).

### Analysis of cell migration by wound-healing assay

The anti-migratory activity was determined with the *in vitro* scratch wound-healing assay. SUIT-2 cells were seeded in 96-well flat bottom plates, at the optimal density of 5 × 10^4^ cells/well in 100 µL and incubated for 24 h. The scratch was performed with a 96-pin scratcher on confluent cell monolayers. After the removal of detached cells, the plate was washed twice with 200 µL of PBS and 100 µL of medium was added to all the wells. Thereafter, the experimental wells were treated with 100 µL of the drugs at concentrations of 4 × IC_50 _and an additional 100 µL of the medium was added to the control wells. Images were taken immediately after scratching procedure, as well as 8 and 24 h after the exposure of the drugs by phase-contrast microscopy using the Leica DMI300B microscope (Leica Microsystems, Eindhoven, the Netherlands). The results were analysed with Scratch Assay 6.2 software (Digital Cell Imaging Labs, Keerbergen, Belgium), as described previously^[53]^.

### PCR assay to evaluate SF3B1 and hENT1

Real-time quantitative reverse transcription PCR (qRT-PCR) was performed to evaluate the gene expression of *SF3B1* and *hENT1 *in the PDAC cell lines, using *GUSB* and *GAPDH* as housekeeping genes. The cells were seeded at 3-5 × 10^3 ^in a 6-well flat bottom plate with 2 mL medium per well and incubated with gemcitabine (IC_50_) for 24 h. Thereafter, the medium was collected and cells were washed using 2.5 mL PBS. Trypsin-EDTA was then added, and, after 5 min incubation the detached cells were resuspended in the previously collected medium and centrifuged at 1500 rpm for 5 min. The pellets were either stored at -80 ℃ or used immediately for RNA extraction, using the RN-easy RNA isolation kit (Qiagen) following the manufacturer’s instructions. One microgram of RNA was then used to synthesise complementary DNA (cDNA) in a volume of 20 µL of sterilised dH_2_O (Versilene® Fresenius, Fresenius Kabi France) for each sample, as described previously^[55]^. The resulting cDNA was amplified by quantitative-PCR using specific primers for SF3B1 and GUSB with the LightCycler® 480 Real-Time PCR System (Roche, Rotkreuz, Switzerland). The mRNA expression of hENT1 was evaluated using the specific kits for hENT1 and GAPDH with the ABIPRISM-7500 instrument (Applied Biosystems, Foster City, CA), as described previously^[41]^.

To visualise the splicing modulation induced by the potential SF3B1 inhibitors on RON, we performed an end-point PCR assay followed by agarose gel electrophoresis. The SUIT-2 cells were seeded in 6-well flat bottom plates and incubated for 24 h with 20 µM of the two most promising compounds in 2 mL medium. RNA isolation and cDNA synthesis were performed according to the protocol described above. The primers for RON were designed considering the exons of this gene, as follows: Exon 10_ Forward (5’-CCT GAA TAT GTG GTC CGA GAC CCC CAG-3’); Exon12_ Reverse (5’-CTA GCT GCT TCC TCC GCC ACC AGT A-3’). PCR was performed as described previously^[55]^, at the annealing temperature of 62 ℃.

### Analysis of antitumour activity in multicellular spheroids of primary cells

PDAC-3 spheroids were established seeding 20000 cells/mL in DMEM/F12 + GlutaMAX-I (1:1), in 24-well ultra-low attachment plates (Corning, NY, USA) according to manufacturers’ protocol. Spheroids were generated for 3-7 days, and then harvested for growth inhibition studies in 96-well plates. After checking their growth rate and stability, the spheroids were treated at concentrations of 4 × IC_50_ of gemcitabine, IS4 and their combination for 72 h. The cytotoxic effects were evaluated by measuring the size of spheroids compared to untreated controls, as described previously^[38]^.

### Statistical analysis

All experiments were performed in triplicate and repeated at least twice. The percentages of cell migration were calculated taking into account at least nine scratches. Data were expressed as mean values ± SEM and analysed by Student’s *t*-test or one-way ANOVA. The cut-off level of significance was *P *< 0.05.

## RESULTS

### Selection of potential SF3B1 inhibitors

**Figure 1:**
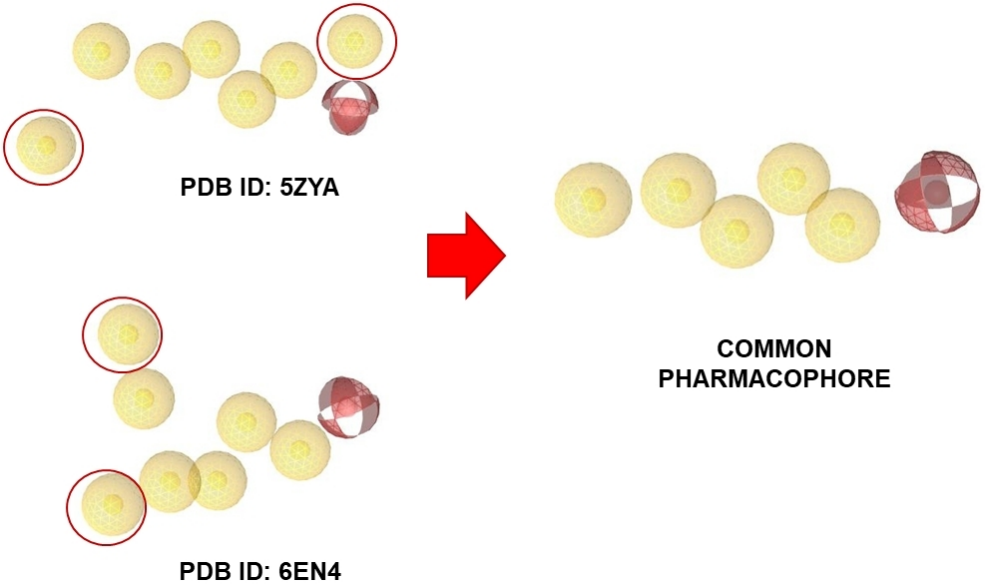
Creation of the common pharmacophore. The common pharmacophore was created starting from the interaction map of the two protein-ligand complexes interacting with PB and E7107, using LigandScout v.4.4. Geometrically common features were selected and six common pharmacophoric features were found.

To explore the binding mode and prioritise putative active compounds, preliminary computational studies were performed using the crystallographic structures of SF3B1 selected from the PDB. The crystallographic structure of two SF3B1 protein ensembles (PDB ID: 6EN4^[56]^ and PDB ID: 5ZYA^[57]^) in complex with PB and its analogue E7107 were selected as a starting point for computational studies to build a structure-based pharmacophore and docking model^[56]^*. *The interaction map of the two protein-ligand complexes was compared as a guide for the crucial interactions to be accounted in our investigations. From the structural analysis of the two compounds compared, the common residues of the protein complex interacting with PB and E7107 were: V1078, V1110, V1114 and L1066 of the subunit SF3B1 and R38 and Y36 in the PHF5A subunit^[57]^. Starting from the two crystal structures, a pharmacophore map was created using LigandScout v.4.4^[46,47]^, and geometrically common features were selected, thus removing two distal features. As a result, six common pharmacophoric features were found and the common pharmacophore was created [Figure 1]. The common pharmacophore was then used for virtual screening studies to identify the molecular scaffolds of interest using both our in-house molecular library and commercially available molecular libraries.

**Figure 2:**
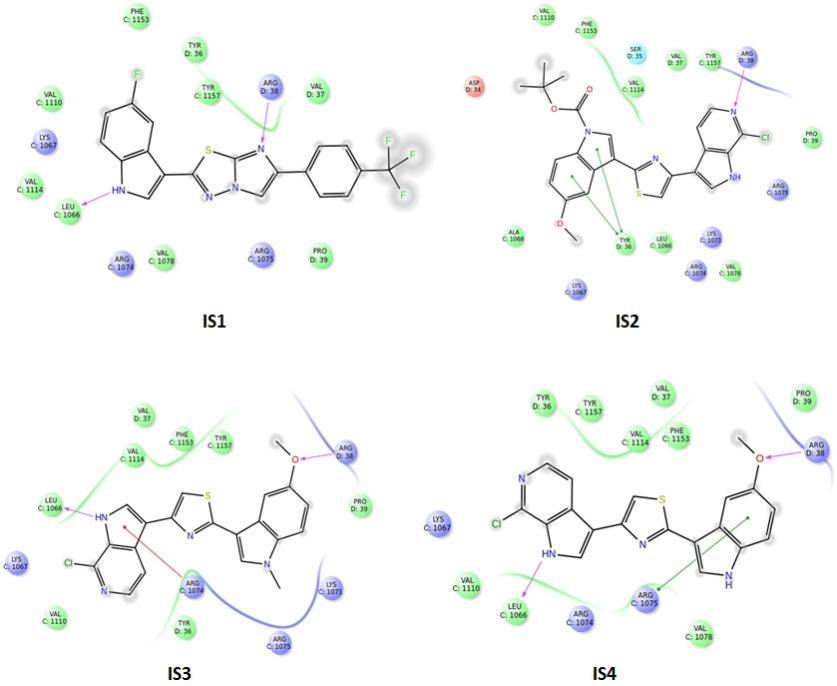
Structure-based pharmacophore and docking were used to prioritise molecules. Tyr36, Arg38, Arg1074, Arg1075 and Leu1066 residues were found to be important for the protein-ligand complex stabilisation. From these analyses, four compounds were prioritised in terms of interactions and theoretical binding energy.

According to the binding mode with the amino acid residues of the common pharmacophore, the most suitable molecules were selected and then their structures were carefully analysed. It was then found that most of them showed a common feature: an indole group and a nearby amide group. Docking studies were performed on the same crystallographic structures using Glide 2018-4^[48]^ to have a consensus mode of selection. Structure-based pharmacophore and docking exploit different algorithms; thus, we decided to see which molecules of our in-house library were prioritised by the two techniques adopted. As shown in Figure 2, Tyr36, Arg38, Arg1074, Arg1075 and Leu1066 residues were found to be important for the protein-ligand complex stabilisation. From these analyses, four compounds were prioritised in terms of interactions and theoretical binding energy.

### Drug sensitivity

**Figure 3:**
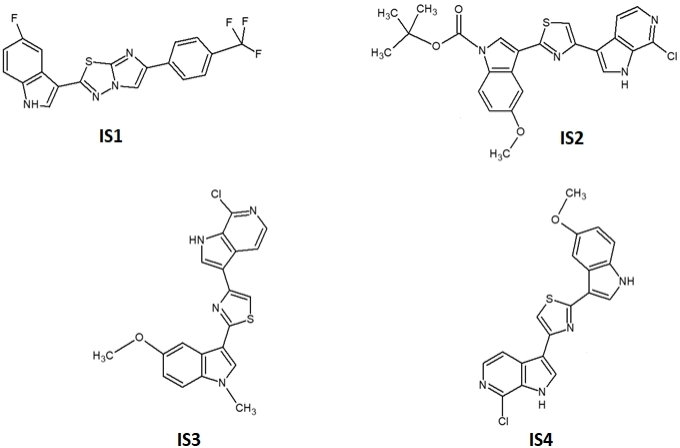
Chemical structures of compounds IS1, IS2, IS3 and IS4. The synthesis of compound IS1 is described in^[39]^, while the descriptions of compounds IS2, IS3 and IS4 can be found in^[49]^.

The *in vitro* sensitivity to the potential SF3B1 modulators {splicing inhibitors IS1, IS2, IS3 and IS4 [Figure 3]} was evaluated for the PDAC cells SUIT-2 and Hs766t. These cells were selected because they are representative of PDAC mesenchymal and epithelial phenotype^[25]^.

**Figure 4:**
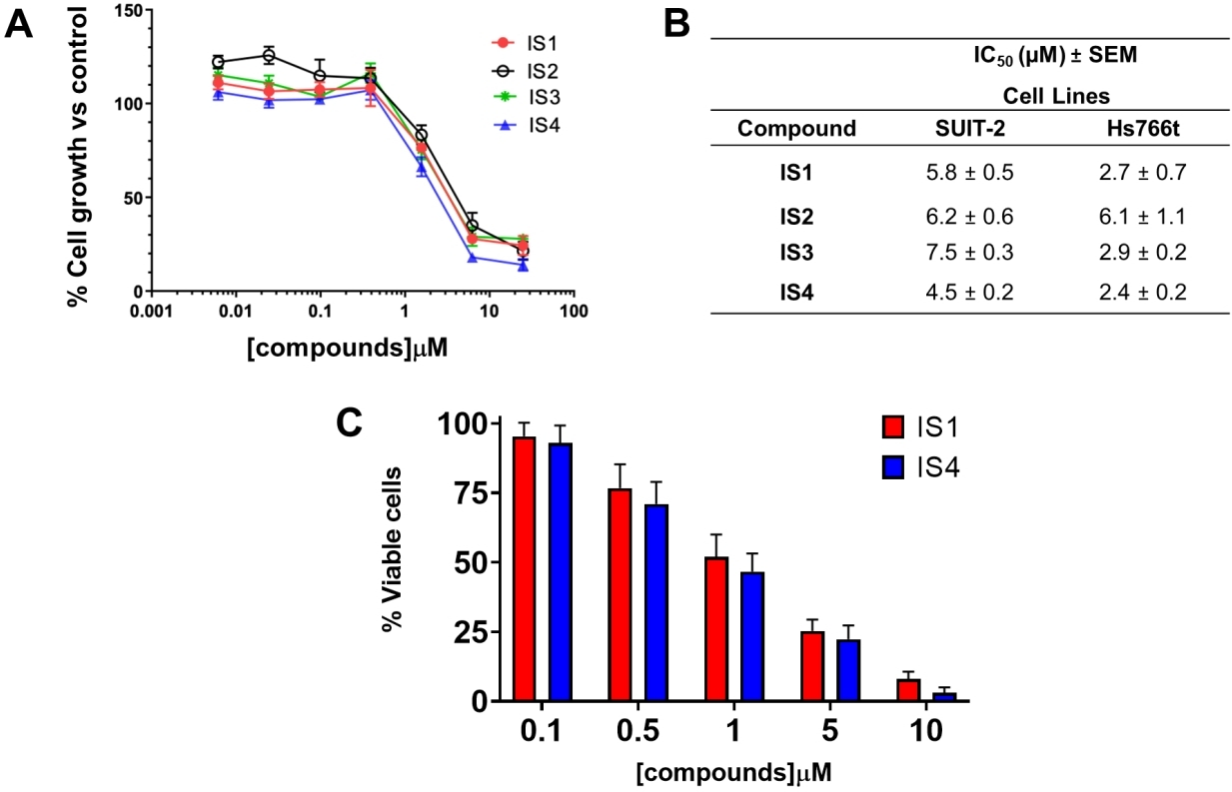
Antiproliferative activity and induction of cell death by IS1 and IS4 in PDAC cells. (A) Representative growth inhibition curves of PDAC cells Hs766t treated for 72 h with the compounds IS1, IS2, IS3 and IS4. Points, mean values; bars, Standard Error of the Mean (SEM). (B) Table summarising the IC_50_ values of the IS1, IS2, IS3 and IS4 compounds against the PDAC cells SUIT-2 and Hs766t. The values are reported as means ± SEM of three separate experiments. IS1 and IS4 had the lowest IC_50_ values and statistical analyses showed significant differences of these compounds compared to both IS2 and IS3. Thus, we selected these compounds for the following studies. (C) Representative bar graph of trypan blue exclusion assay, showing the percentage of viable Panc05.04 cells after treatment for 96 h and the compound IS1 and IS4 at five different concentrations (0.1, 0.5, 1, 5 and 10 µM). Columns, mean values; bars, SEM.

A pre-screening cytotoxicity SRB assay was initially performed using concentrations of 0.1, 1 and 10 µM. Notably, all compounds showed concentration-dependent inhibition of proliferation; thus, we expanded our studies using at least eight different concentrations (from 125 nM to 16 µM) to define more accurate IC_50_ values. Compounds IS1 and IS4 showed the highest sensitivity in both preclinical models [Figure 4A and B]. In particular, the Hs766t cells were most sensitive to both compounds, with IC_50_s of 2.4 and 2.7 µM after exposure to IS4 and IS1, respectively. In contrast, the SUIT-2 cells were least sensitive, with IC_50_s ranging from 4.5 to 7.5 µM. Considering the interesting results of antiproliferative activity *in vitro*, we selected the most promising compounds (IS1 and IS4) for the analysis of migration inhibition and the modulation of the splicing of RON, an overexpressed gene in PDAC.

### Induction of cell death in cells harbouring SF3B1 mutations

Heterozygous mutations in the splicing factor *SF3B1* have been found to particularly occur in haematological malignancies, but more recently they have also been detected in several solid tumours including PDAC^[41]^ with a frequency of 4%^[58-60]^. Previous studies have shown that *SF3B1 *mutations are concentrated in the sequence encoding the HEAT repeat domains with major hotspots including p.R625, p.K666 and p.K700E^[60-62]^. Interestingly, the latter mutation is carried by the Panc05.04 cell line together with p.Q699H^[63]^. Therefore, we used this model to perform further studies with the IS1 and IS4 compounds. Notably, the mutations of SF3B1 do not affect SF3B1 gene expression, which is similar to the other PDAC cells, as assessed by PCR (data not shown).

Panc05.04 cells have a relatively long doubling time (46 h) compared to most ATCC cell lines. To achieve reliable results, these cells were exposed for 96 h to compounds IS1 and IS4, at five different concentrations in the micromolar range (from 0.1 to 10 µM). Remarkably, both drugs induced cell death, ranging from 52% to 63% at a concentration of 1 µM [Figure 4C]. However, since these Panc05.4 cells are the only known PDAC cells harbouring a mutation in SF3B1, we could not draw conclusions on whether they are more sensitive to potential SF3B1 inhibitors.

### Anti-migratory activity and modulation of RON splicing pattern

**Figure 5:**
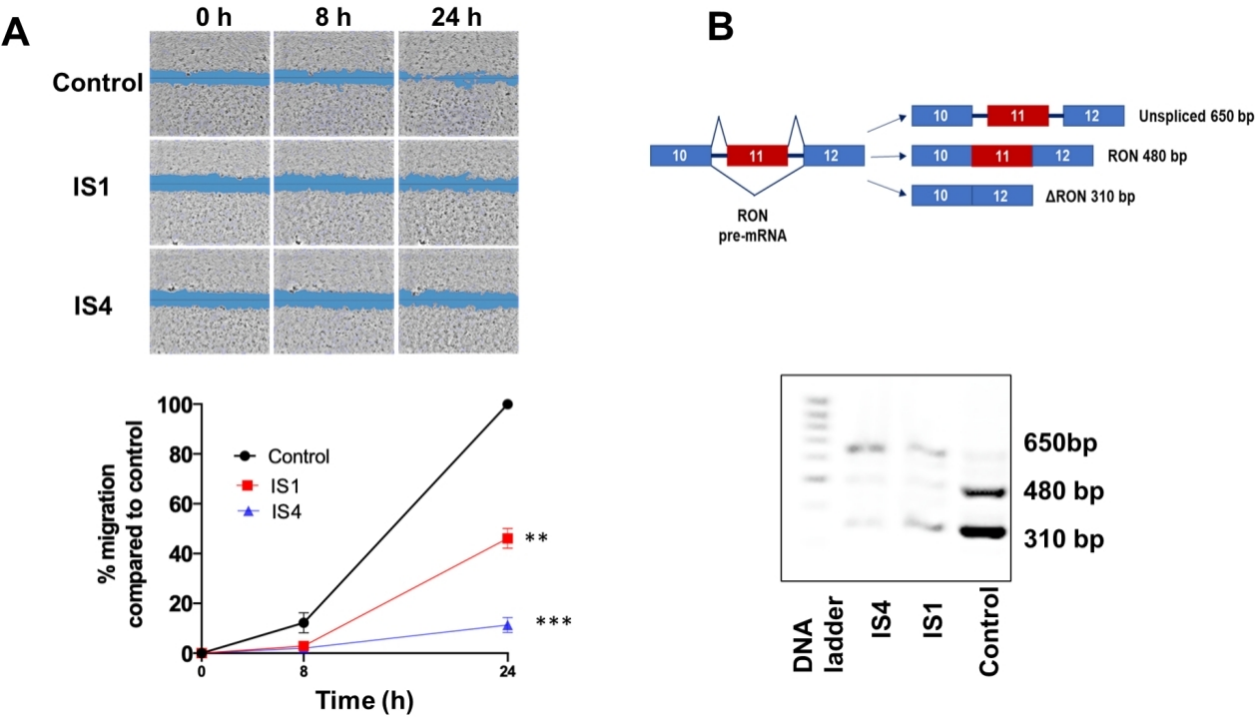
Inhibition of migration and modulation of RON splicing by compounds IS1 and IS4 in SUIT-2 cells. (A) (Top) Representative pictures of scratch areas in untreated (control) and treated cells during the wound healing assay. Original magnification 5×. (Bottom) Percentage of migration over time (0, 8 and 24 h) of SUIT-2 cells treated with the compounds IS1 and IS4 at concentrations of 4 × IC_50_. **P < 0.01, ***P < 0.005. (B) (Top) Schemes of RON pre-mRNA variant structures with predicted PCR products are shown on the left. (Bottom) Representative picture of PCR splicing pattern assessment of RON in SUIT-2 cells after 24 h of drug exposure. Original magnification 5×.

The metastatic potential is one of the hallmarks of PDAC, and it is closely related to the grim prognosis of this disease. Currently, the key mechanisms underlying this process are poorly understood, although it has been shown that several factors govern the metastatic process, including cell migration and invasion^[5]^. The promising results on the antiproliferative activity prompted us to also investigate the anti-migratory effect of our potential SF3B1 modulators by the wound healing assay, which was performed on SUIT-2 cells. These cells were selected because of their ability to form monolayers at optimal cell confluence within 24 h. A concentration of 4 × IC_50_ was used for each compound because of the shorter drug exposure time compared to growth inhibition studies, which lasted 72 h, and because it was able to slightly reduce migration already after 8 h exposure compared to untreated cells (set at 100%). However, IS1 and IS4 significantly inhibited the migration rate of SUIT-2 cells after 24 h of drug exposure [Figure 5A], with percentages of migration rates below 40% and 10% for IS1 and IS4, respectively.

Remarkably, this effect was associated with the mis-splicing of RON, which is a tyrosine kinase receptor belonging to the c-MET kinase family. This gene is overexpressed in PDAC and promotes cell migration, invasion and apoptotic resistance^[64,65]^. Of note, RON commonly undergoes AS resulting in different shorter ΔRON spliced variants^[66]^. The PDAC SUIT-2 cells express the truncated variant ∆RON, which plays a pivotal role in tumour cell motility due to the constantly activated kinase function^[65]^. A 24 h exposure to IS1 and IS4 caused intron retention in RON transcript and decrease in transcript abundance, probably due to nonsense-mediated decay [Figure 5B].

### Synergistic interaction with gemcitabine is associated with an increase of hENT1 mRNA expression

Gemcitabine is a pyrimidine analogue (2’,2’-difluoro-2’-deoxycytidine, dFdC; Gemzar®) widely prescribed to treat a variety of solid tumours^[67]^. It has been used for decades as the first-line treatment for metastatic PDAC, and it is still commonly used for PDAC patients in combination with nab-paclitaxel or as monotherapy in patients who are unfit for combination regimens, as mentioned above^[7,9]^.

Our previous data show that some compounds from a series of new imidazo[2,1-*b*][1,3,4]thiadiazole derivatives potentiated the antiproliferative effects of gemcitabine in peritoneal mesothelioma cells^[40]^. However, different splicing aberrations have previously been shown to enhance the activity of proliferative and glycolytic signalling associated to gemcitabine resistance^[68-70]^.

**Figure 6:**
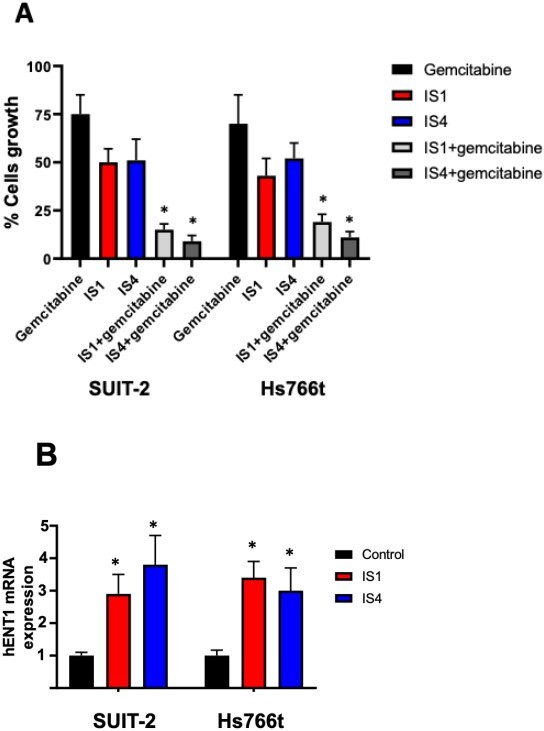
Increase of gemcitabine sensitivity. (A) Effect on growth of SUIT-2 and Hs766t cells of the combination of gemcitabine at its IC_25_, with the compounds IS1 and IS4 at their IC_50_. The observed values were lower than the theoretical values. (B) Modulation of hENT1 mRNA levels. Expression was determined with quantitative-PCR by normalisation with the GAPDH housekeeping gene, as described in the methods. Since we previously demonstrated that hENT1 protein levels correlated with hENT mRNA expression, we did not include hENT1 protein expression^[71,72]^. Columns, mean values obtained from triplicate experiments; bars, SEM; *P < 0.05.

Therefore, we evaluated whether the combinations with the compounds IS1 and IS4 at their IC_50_ would increase sensitivity to gemcitabine of SUIT-2 and Hs766t cells. The combination of both compounds IS1 and IS4 with gemcitabine at IC_25_ levels led to a significant reduction in cell growth compared to untreated cells, below 20% and 12%, respectively [Figure 6A]^[71,72]^. These values were well below the theoretical achievable growth inhibition of the combinations and can therefore be considered as a synergistic effect.

Because of its hydrophilic nature, gemcitabine requires facilitated or active transport for cellular uptake, which is mediated by membrane nucleoside transporters, including the human concentrative nucleoside transporter-3 and hENT1. The latter has been evaluated in several preclinical and clinical studies as a potential determinant of gemcitabine efficacy in PDAC^[9]^.

Previously, our imidazo[2,1-*b*][1,3,4]thiadiazole compounds in combination with gemcitabine significantly increased the expression of hENT1, suggesting its potential role in increasing the activity of gemcitabine^[40]^. These promising results prompted us to adopt the same strategy to investigate potential molecular mechanisms underlying the reduced activity of gemcitabine in combination with IS1 and IS4. Therefore, we measured the modulation of the gene expression of hENT1. Both compounds, also in this case, increased hENT1 expression significantly [Figure 6B], supporting the role of these new compounds in reversing a key mechanism of resistance to gemcitabine.

### The combination of gemcitabine and IS4 reduced spheroids of PDAC primary cultures

**Figure 7:**
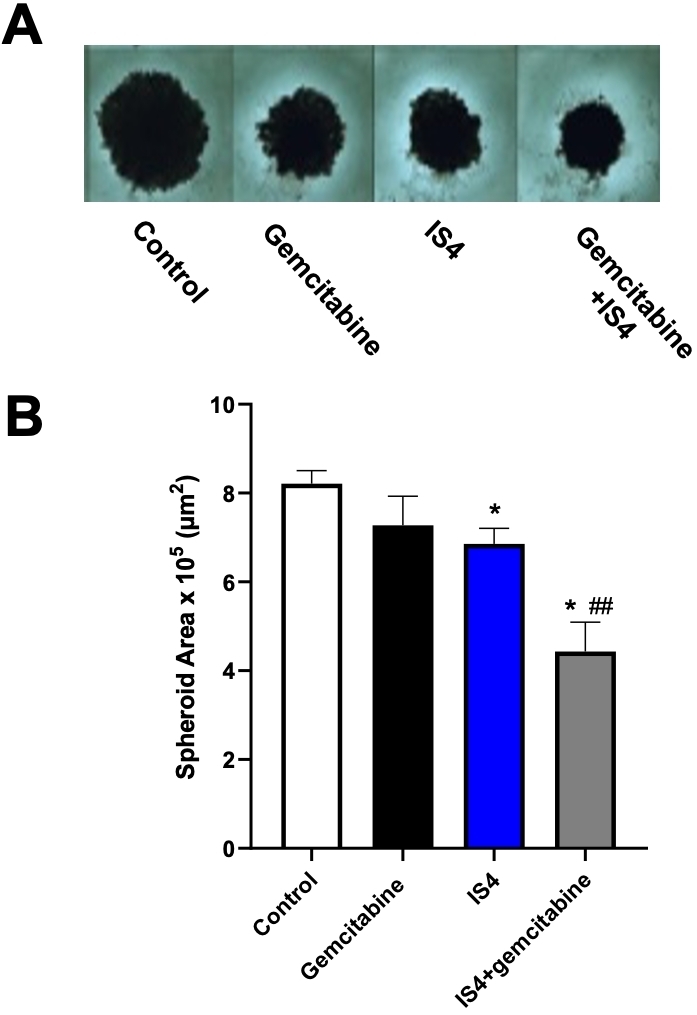
Increase of gemcitabine sensitivity in 3D models. (A) Representative images of PDAC-3 spheroids untreated (control) or treated with gemcitabine, IS4 or their combination (original magnification, 40×). (B) Effects of gemcitabine, IS4 or their combination on the areas of PDAC-3 spheroids after 72 h exposure. Columns, mean values obtained from triplicate experiments; bars, SEM; *P < 0.05 *vs*. control, ^##^*P *< 0.01 *vs*. gemcitabine.

The sensitivity to anticancer drugs, including gemcitabine, in two-dimensional monolayer cell culture models is typically different from three-dimensional (3D) culture models. Thus, to determine whether IS4 would enhance the efficacy of gemcitabine in 3D systems, we tested these drugs in spheroids of PDAC3 cells [Figure 7A]. We transferred in each well of 96-well plates spheroids that were approximately 500 μm in diameter. These growing spheroids were exposed to gemcitabine, IS4 and their combination for 72 h. The growth of these spheroids was slightly inhibited by gemcitabine and IS4, while the combination remarkably increased their disintegration, and they were significantly reduced in size compared to the untreated spheroids as well as to spheroids exposed to gemcitabine-alone [Figure 7B].

## DISCUSSION

In this paper, we demonstrate that in PDAC cells inhibition of splicing can help to fight the typical resistant behaviour of these tumours to standard chemotherapeutic drugs, such as gemcitabine, most likely by reducing cell aggressiveness/invasiveness and increasing the expression of the limiting uptake transporter hENT1. The treatment of patients with gemcitabine alone gives a moderate effect, and any improvement of this effect would increase the prospects of PDAC patients. Only 15%-20% of all PDAC patients qualify for curative resection followed by adjuvant chemotherapy, often including gemcitabine^[5]^, and treatment options for most PDAC patients are limited. Thus, there is a clear need for new therapeutic approaches targeting key determinants of PDAC aggressive behaviour and reversing or bypassing resistance to existing therapies^[10]^.

Recent genomic studies have shown that heterozygous mutations in the splicing factor SF3B1 frequently occur in several tumours and prompt cancer progression through the activation of cryptic splice sites in multiple genes^[11]^. Most SF3B1 mutations have been detected in haematological malignancies, but PDAC is among the solid tumours harbouring these mutations in more than 3% of cases^[12,59]^. Moreover, PDAC has high levels of expression of SF3B1, and recent studies have demonstrated a positive correlation between expression levels of wildtype (WT) SF3B1 and tumour malignancy^[11,62]^, further supporting the search for drugs targeting this key spliceosomal factor.

In the present study, we evaluated for the first time four potential spliceosome inhibitors {one imidazo[2,1-*b*][1,3,4]thiadiazole derivative (IS1) and three indole derivatives (IS2, IS3 and IS4)}, which were selected by virtual screening from an in-house molecular library in order to investigate their potential efficacy against PDAC cells. Similar approaches have allowed identifying several splicing modulators other than SF3B1 inhibitors in different high-throughput screens, which are currently undergoing further evaluation in preclinical studies, as reviewed previously^[73,74]^.

The emerging potential SF3B1 modulators IS1 and IS4 were able to inhibit cell proliferation in SUIT-2 and Hs766t cells, displaying IC_50_ values ranging from 2.4 to 5.8 µM. Remarkable growth inhibition was also observed in Panc05.04 cells, harbouring SF3B1 mutations. This is in agreement with previous findings, showing that E7107 substantially reduced leukaemia cell burden in an isogenic mouse model carrying an Srsf2 P95H mutation as well as in PDX models from patients harbouring SRSF2 mutations compared to WT models^[75]^.

The IC_50_ values observed after treatment with our most promising compounds were however higher than what has been reported for PB and E7107 in different preclinical models of solid tumours, such as mesothelioma, where IC_50_ values of these SF3B1 modulators are in the nanomolar range^[54]^. However, this might mitigate adverse events, which limited the clinical development of E7107^[19]^. Similarly, the excellent results of splice-switching oligonucleotides and RNA interference *in vitro* are extremely difficult to translate to the clinical setting due to limited stability in plasma and intracellular uptake^[76]^.

In the present study, we also evaluated the modulation of the gene expression of hENT1. It has been reported repeatedly that high hENT1 levels are correlated with increased gemcitabine cytotoxicity and prolonged disease-free status and overall-survival in patients receiving gemcitabine adjuvant chemotherapy^[41]^, including a PCR on laser-microdissected tissues study in which Giovannetti *et al*.^[77]^ reported an overall survival of 25.7 and 8.5 months in PDAC patients with high and low levels of hENT1, respectively. Of note, the expression and activity of hENT1 is affected by multiple molecular mechanisms. In particular, it is worth mentioning that the TME of PDAC influences the expression of hENT1 causing PDAC gemcitabine chemoresistance. In fact, various components of the extracellular matrix limit the availability of oxygen (hypoxia), hindering the transport of gemcitabine via hENT1^[41]^. Of note, several polymorphisms may affect the gene expression of hENT1, and therefore the efficacy of gemcitabine. Specifically, Myers and collaborators showed that individuals with CAG and CGC haplotypes exhibited significantly higher hENT1 expression than individuals with the normal CGG haplotype^[78]^. Other mechanisms affecting hENT1 expression include epigenetic modulation and microRNA^[41]^, and recent studies have shown interesting interrelationships between miRNA and splicing factors in PDAC^[79]^.

Remarkably, the IS1 and IS4 compounds potentiated the activity of gemcitabine. In previous studies, after SF3B1 and PHF5A knockdown, leukaemia cells became highly sensitive to mitomycin C, suggesting that a combination of splicing modulation with DNA damaging agents could achieve synergistic effects^[80]^. However, we might also hypothesise that this effect is caused by the positive modulation of hENT1 mRNA expression, for which a low expression has been associated with gemcitabine resistance in different cancer cell types^[41]^. Thus, our data suggest that splicing inhibition can reverse resistance to gemcitabine.

In addition, using a 3D culture model (e.g., spheroids) of primary cell culture that mimics the 3D organisation of PDAC tumour cells *in vivo*^[81]^, we showed that the antitumour activity of gemcitabine was significantly increased by the simultaneous addition of IS4.

Finally, the IS1 and IS4 compounds were also able to induce a splicing shift from RON and ΔRON after 24 h from the start of treatment, which might at least in part explain the strong anti-migratory ability of IS1 and IS4 in SUIT-2 cells. Of note, RON and cMET are important indicators of prognosis in PDAC, and previous studies have shown the synergistic interaction of inhibitors of these protein kinases with gemcitabine^[81,82]^, further providing new means to predict clinical outcome and targets for more effective therapies against PDAC.

Other markers should be evaluated in the future. However, another splice variant evaluated in previous studies, MCL-1 (myeloid cell leukemia 1)^[58]^, did not show an aberrant splicing pattern when evaluated using IS4 and not even with PB as reference splicing inhibitor. Therefore, we did not proceed with this marker in view of our potential SF3B1 modulators.

Novel compounds targeting pivotal splicing factors, such as SF3B1, could have relevant antitumour activity, and, in the present study, we identified four potential SF3B1 inhibitors, selected from an in-house library, that showed cytotoxic and antimigratory activity in PDAC cells and potentiated the antitumour effects of gemcitabine. Our studies supported the role of RON and hENT1 modulation as molecular mechanisms to be further exploited for the characterisation of these new therapeutic approaches, other than for prognostic purposes^[1]^.

In conclusion, our novel findings prompt further analysis of the selectivity and toxicity of our potential SF3B1 inhibitors, as well as the role of the modulation of RON and hENT1 for further studies in appropriate preclinical models, including *in vivo* models and new model systems^[83]^, in order to guide the rational development of new drug combinations that could reverse chemoresistance of PDAC.

## DECLARATIONS

### Copyright

© The Author(s) 2021.
